# Concordance of HIV Prevention Advocacy Reports and its Associations with HIV Protective Behaviors

**DOI:** 10.1007/s10461-024-04412-0

**Published:** 2024-06-20

**Authors:** Nipher Malika, Harold D. Green, Laura M. Bogart, Joseph K.B. Matovu, David J. Klein, Steven Okoboi, Violet Gwokyalya, Susan Ninsiima, Glenn J. Wagner

**Affiliations:** 1 RAND Corporation, 1776 Main St, Santa Monica, CA 90401 USA; 2 Department of Applied Health Science, Indiana University School of Public Health- Bloomington, Bloomington, IN USA; 3 Makerere University School of Public Health, Kampala, Uganda; 4 Infectious Disease Institute, College of Health Sciences, Kampala, Uganda

**Keywords:** HIV, Prevention advocacy, Social networks, Network-based interventions, Peer-to-peer interventions

## Abstract

Peer advocacy can promote HIV protective behaviors, but little is known about the concordance on prevention advocacy(PA) reports between people living with HIV(PLWH) and their social network members. We examined prevalence and correlates of such concordance, and its association with the targeted HIV protective behavior of the social network member. Data were analyzed from 193 PLWH(index participants) and their 599 social network members(alters). Kappa statistics measured concordance between index and alter reports of PA in the past 3 months. Logistic and multinomial regressions evaluated the relationship between advocacy concordance and alter condom use and HIV testing behavior and correlates of PA concordance. Advocacy concordance was observed in 0.3% of index-alter dyads for PrEP discussion, 9% for condom use, 18% for HIV testing, 26% for care engagement, and 49% for antiretroviral use discussions. Fewer indexes reported condom use(23.5% vs. 28.1%;${ \chi }^{2}$=3.7, *p*=0.05) and HIV testing(30.5% vs. 50.5%; ${\chi }^{2}$=25.3, *p*<0.001) PA occurring. Condom advocacy concordance was higher if the index and alter were romantic partners(OR=3.50; *p*=0.02), and lower if the index was 10 years younger than the alter(OR=0.23; *p* = 0.02). Alters had higher odds of using condoms with their main partner when both reported condom advocacy compared to dyads where neither reported advocacy(OR=3.90; *p*<0.001) and compared to dyads where only the index reported such advocacy(OR = 3.71; *p*=0.01). Age difference and relationship status impact advocacy agreement, and concordant perceptions of advocacy are linked to increased HIV protective behaviors. Alters’ perceptions may be crucial for behavior change, informing strategies for improving advocacy.

## Introduction

HIV prevention advocacy plays a pivotal role in raising awareness, combating stigma, and ensuring access to vital resources for those impacted by HIV. The influence of prevention advocacy extends beyond mere awareness-raising; it serves as a catalyst for policy shifts, financial allocations, and community initiatives aimed at prevention, treatment, and support services [1–4]. Advocacy not only meets immediate needs but also propels broader public health objectives, striving to curtail incidence rates, foster preventive behaviors, safeguard the well-being of affected communities, and positively shift societal perceptions to be less stigmatizing toward people affected by HIV [4–7]. 

Peer-based advocacy interventions have emerged as powerful and effective approaches to promote HIV protective behaviors in diverse populations [8]. In countries like Uganda, for instance, where the HIV prevalence rate is 6.2% and the incidence rate at 2.2% has stagnated [9–11], peer-based approaches offer promising avenues to bolster adherence to antiretroviral therapy (ART), ensure sustained retention in HIV care [12, 13], reduce condomless sex [14], and increase HIV testing [15, 16]. To comprehensively assess the effectiveness of peer-based interventions, gaining a deeper insight into the agreement between advocates and recipients regarding the occurrence of advocacy may be crucial. Advocacy operates within a relational, dyadic structure involving an advocate and a recipient entity or group. Advocacy is not solely about message delivery; understanding how advocacy is received and interpreted is pivotal for evaluating its impact. However, we are unaware of any published study that has examined concordance within dyads regarding the presence of HIV prevention advocacy.

Most HIV concordance studies often focus on serostatus concordance and/or prevention behaviors such as condom use and voluntary counseling of sexual partners [17–20]. On a broader scope, non-HIV studies exploring concordance focus on various aspects like health reporting, personality traits, and sexual behavior within couples [21–24]. However, none of these studies focus on concordance of perception and its correlation to behavior change.

Although evaluations of peer-based HIV prevention interventions have been demonstrated to affect substantial behavioral changes [25–27], they often fall short in exploring the inherent relationship within the broader range of dyads in which advocacy might occur and whether the members of dyad are concordant in their perception that advocacy occurred. Questions remain, including: what characteristics of the advocate/recipient dyad are associated with concordance of reported advocacy, and is there a noticeable behavioral change related to prevention advocacy concordance within dyadic relationships?

The dyadic health influence model [28] can help to explain why concordance in perceptions between members of a dyad can affect health behaviors. This model suggests that intentional strategies such as advocacy can influence an alter’s health behavior if there is agreement between the index (the focal individual within the network analysis) and the alter (the individuals directly connected to the index within the network) that advocacy occurred [28]. The dyadic health influence model outlines three pathways for one person (e.g., the index) to influence the health beliefs and actions of their partner (e.g., an alter). First the index can lead by example by showcasing healthy behaviors and creating a supportive environment. Second, they might engage in behaviors that benefit the dyadic relationship, thereby indirectly impacting the alter’s health actions. And lastly, the index might intentionally employ strategies, like advocacy, to directly influence the target’s health behavior. This model underscores that the index’s actions are rooted in their perception of the alter’s health and the dynamics within their relationship; their behaviors affect not just the health choices of the alter but also the dynamics within their relationship.

Using the dyadic health influence model as our framework, the present study had three specific objectives: (1) to assess concordance in reports of HIV prevention advocacy between index PLWH and their alters in a study in Kampala, Uganda, and (2) to identify factors associated with index-alter prevention advocacy concordance, and (3) to investigate whether index-alter concordance regarding prevention advocacy correlates with alter use of HIV protective behavior.

## Methods

### Study Setting

All study activities were conducted in Kampala, Uganda at the Infectious Disease Institute (IDI). IDI provides outpatient care to approximately 8,000 active HIV-positive patients and conducts HIV-related clinical trials. This study received IRB approvals from IDI, and the Human Subjects Protection Committee of the RAND Corporation. All participants provided written informed consent.

### Participants and Procedures

Data were from a baseline sample of 193 PWH (indexes) and 599 alters from the indexes’ social networks [29]. During the recruitment period, eligible IDI patients were referred to interviewers by clinic staff and expert patients (individuals who volunteer and collaborate with clinic staff. They are familiar with clinic procedures, skilled in patient counseling, and often have strong relationships with patients, leading to higher levels of trust due to their relatability). Eligible indexes were ≥ 18 years old, in care for > 1 year, and patients at IDI’s clinic. Clients were excluded if they participated in the intervention pilot study [30]. 

Each patient participant was asked to identify 20 alters in their social network who knew their serostatus. At the end of the index recruitment interview, indexes were asked to select, from the list of alters who knew their serostatus, up to 7 alters whom they would be willing to refer to the study. If they referred more than 4 alters, we randomly selected 4 to target for recruitment (to limit selection bias). Alters were recruited in 4 waves and each wave recruitment took 8 weeks; the average number of social network members recruited was 2.8 (SD = 1.53, range = 0–7). (Midway through the study, we allowed more than four alter recruits to help us reach our target sample size). Baseline data collection started January 2022 and ended February 2023. Eligible alters were ≥ 18 years-old, referred by an index enrolled in the study, aware of the index’s HIV serostatus, and willing and able to do the baseline survey in person. Our participants’ sociocultural context is shaped by several factors. Firstly, while Uganda has made strides in increasing access to antiretroviral therapy, stigma and discrimination against people living with HIV/AIDS persist, impacting their social interactions, employment opportunities, and mental health. Secondly, traditional beliefs and practices influence perceptions of education, health, and gender roles. For example, some cultural practices may hinder women’s access to education or stigmatize HIV-positive individuals. Gender inequality remains a challenge, affecting women’s education, health outcomes, and participation in the workforce. Lastly, although literacy rates in Uganda have improved, there are notable disparities based on gender and geography.

### Assessment

The assessment was interviewer-administered and conducted in Luganda. All measures were developed by the study team and had been translated from English to Luganda using a standard translation/backtranslation methodology from a prior study [21]. Participants received 30,000–70,000 Uganda shillings (~$8–20 USD), depending on the distance traveled, to cover transportation costs.

#### Socio-Demographic Characteristics

We assessed self-reported age (in years), gender (male, female, other), education (primary school, secondary school, university level, other), occupation (farmer, salaried, business/sells things, other), income (≤500,000USh, > 500,000USh), and relationship status (single, married, divorced/separated/widowed).

#### Concordance of Perceived Occurrence of HIV Prevention Advocacy

We evaluated prevention advocacy efforts targeted towards enrolled alters by the index participants. Prevention advocacy was defined as “as talking to other people about protecting themselves from HIV (through get tested, safer sex, and taking antiretroviral therapy (ARVs) or Pre-exposure prophylaxis (PrEP)).” If such discussions were reported, we then asked follow-up questions specifically related to advocacy, such as whether the conversation included encouragement or information aimed at promoting testing/condom use, in order to ascertain the genuine presence of advocacy. For each alter, the index participant was asked whether they had discussed condom use, HIV testing, PrEP (if the alter was not HIV-positive), and engagement in HIV care and, if the alter was HIV-positive, use of ARV (if the alter was HIV-positive), in the last three months. The index responded either “yes” or “no” to each question. Similarly, each alter was also asked to respond “yes” or “no” to whether their index had discussed each of these HIV protective behaviors, in separate items.

#### Dyadic Network Characteristics

The index was asked whether they trust the alter (yes/no), the alter’s relationship with the index (parent/grandparent/caregiver, sibling, child, other relative, friend, spouse/main partner) and frequency of contact (never, 1–3 times/month, 1–2 times/week, 3–5 times/week, everyday).

#### HIV Protective Behaviors

These included alter condom use (“yes”, they used condoms the last time they had sexual intercourse with their main partner or spouse vs. “no” they did not use condoms during this last sexual encounter) among all alters, and HIV testing in the last 6 months (“yes”, they tested vs. “no” they haven’t tested) among those who did not report testing HIV-positive.

### Statistical Analysis

Descriptive statistics were used to characterize the characteristics of the sample.

#### Concordance Analysis

We report the frequency and percent of concordant responses (e.g., both index and alter reported “yes” or both reported “no”) and discordant responses (index reported “yes” and alter partner reported “no” or vice versa) to questions asking whether the index provided prevention advocacy on HIV testing, condom use, engaging in HIV care, taking ARVs and PrEP use.

Kappa statistics are reported to measure concordance of dyadic responses over and above what would be expected by chance alone. Because each index participant talked with multiple alters, kappa statistics were adjusted for clustering using the bootstrap method [31]. In general, values of Kappa from 0 to 0.20 indicate poor agreement, 0.21 to 0.40 indicate fair agreement, 0.41 to 0.60 indicate moderate agreement, 0.61 to 0.80 indicate substantial agreement, and values greater than 0.80 indicate excellent agreement [32]. Kappa values may be underestimated when the prevalence of responses measures is skewed. Therefore, in conjunction with the Kappa statistic, Ochs and Binik [33] recommend running conditional probability indices to explore the degree to which a pair (e.g., index and alter) reporting of data is consistent. A positive conditional probability (CP+) is the average probability that either the index or the alter reports an advocacy event, given that the other partner also reports the advocacy event.

McNemar’s statistic is also provided along with its associated *p*-value assessing whether marginal frequencies are equal. Unlike the Kappa and conditional probability measures, McNemar’s statistic does not address agreement between the index and alters directly, but rather measures agreement in the proportion of individuals reporting advocacy. In this study, McNemar’s statistic reflects the difference between the proportion of indexes who reported “yes” versus the proportion of alters who reported “yes” for each binary outcome of interest. A significant *p*-value associated with the McNemar test indicates a tendency of alters to answer the question differently from indexes, independent of the experience of any given dyad. To account for clustering at the index participant level, Obuchowski’s chi-square statistic was calculated [34, 35]. 

#### Regression Analysis

To understand if index-alter prevention advocacy concordance is associated with alter behavior, we employed clustered binary logistic regression at the index level, using the index-alter dyad as the unit of analysis and adjusting standard errors for clustering at the index level. The dependent variables were alter condom use and HIV testing. Independent variables included dyad gender composition (female index/female alter, male index/male alter, female index/male alter, male index/female alter) and dyad age difference (index is at least 10 years older than alter, index is at least 10 years younger than alter, index is within 10 years of the alter). We employ age categorization spanning 10 + years older, 10 + years younger and within 10 years of the index’s age to explore communication patterns in authority relationships [36]. In addition, considering that certain index-alter relationships may be romantic in nature, exploring HIV risk within age-disparate relationships becomes pertinent, particularly due to the vulnerability young women might face when involved with older male partners. Logistic regressions were performed, each focusing on a specific binary independent variable: (1) concordance reporting of prevention advocacy being present when compared to no reporting of prevention advocacy, (2) concordance reporting of prevention advocacy compared to only index reporting of prevention advocacy, (3) concordance reporting of prevention advocacy when compared to only alter reporting of prevention advocacy, (4) concordance on no prevention advocacy compared to only the index reporting of advocacy, (5) no reporting of prevention advocacy compared to only alter reporting of advocacy, and (6) only index reporting advocacy compared to only alter reporting advocacy.

Finally, we performed a multinomial logistic regression analysis to examine the correlates of a 4-tiered concordance/discordance variable reflecting agreement or disagreement between index and alter regarding whether prevention advocacy occurred: level 0 (reference group) - agreement in no advocacy efforts; level 1 - alter reports advocacy but index does not; level 2 - index reports advocacy but alter does not; level 3 - both index and alter report advocacy occurred. The independent variables included dyad gender composition, age homophily and dyadic variables (i.e., index trust of alter, relationship status and frequency of contact) as predictors. All analyses were conducted using SAS 9.4 version.

## Results

### Demographic Characteristics

Table 1 presents index- and alter-specific demographic characteristics. Index participants were significantly older than alter participants (45.6 vs. 37.7 years; $${\chi }^{2}$$=5.2 *p*<0.001) and index participants were more likely to be single or divorced (single never married 38.8% vs. 33.7%; divorced/separated/widowed 20.7% vs. 15%; $${\chi }^{2}$$=10.3, *p*=0.03), and in business professions compared to alters (61.6% vs. 48.9%, $${\chi }^{2}$$=17.3, *p*=0.04), and were less likely to spend more than 500,000 Ugandan Shillings ($133) per month compared to alters (74.9% vs. 67.1%; $${\chi }^{2}$$=6.7, *p* = 0.01).

**Table 1:** Baseline demographic characteristics

	Index (*n* = 193)	Alters (*n* = 599)	Chi-Square (*p*-value)
Age [M(SD), Range]	45.6 (10.3), 22–69 yrs	37.7 (12.7) 18–81 yrs	5.2 (< 0.0001)
Sex at birth			3.3 (0.07)
Male	59 (30.5%)	202 (33.7%)	
Female	134 (69.4%)	396 (66.1%)	
Other	0 (0%)	1 (0.2%)	
Married/living with significant other			10.3 (0.03)
Single	75 (38.8%)	202 (33.7%)	
Married	78 (40.4%)	307 (51.3%)	
Divorced/Separated/Widowed	40 (20.7%)	90 (15.0%)	
Highest education completed			42.1 (< 0.0001)
Primary School	75 (39.9%)	193 (32.9%)	
Secondary School	93 (49.5%)	273 (46.5%)	
University level	19 (10.1%)	87 (14.8%)	
Other	1 (0.5%)	33 (5.6%)	
Occupation			17.3 (0.04)
Farmer	14 (7.3%)	43 (7.2%)	
Salaried	33 (17.1%)	111 (18.6%)	
Business/sells things	119 (61.6%)	292 (48.9%)	
Other	27 (13.9%)	151 (25.3%)	
Average monthly expenditure			6.7 (0.008)
≤500,000ΥΣη	143 (74.9%)	396 (67.1%)	
> 500,000USh	48 (25.1%)	194 (32.8%)	
HIV status			+
HIV+	193(100%)	207 (35.8%)	
HIV-	0 (0%)	371 (64.2%)	
Length of time with main partner			45.7 (0.0001)
< 6 Months	6 (4.4%)	13 (2.8%)	
6–12 months	5 (3.6%)	32 (6.9%)	
1–2 years	21 (15.3%)	60 (13.1%)	
3-5years	27 (19.7%)	86 (18.7%)	
6 + years	78 (56.9%)	268 (58.4%)	
HIV Disclosure to main partner			0.02 (0.87)
Yes	107 (78.1%)	399 (90.6%)	
No	30 (21.9%)	41 (9.6%)	
Main partner HIV status			4.3 (0.37)
HIV+	53 (43.1%)	121 (28.6%)	
HIV-	65 (52.8%)	292 (69.2%)	
Not Tested	5 (4.1%)	9 (2.1%)	

+Statistics were derived to assess differences between groups. With HIV status, all index participants had to be HIV + to be in the study thus we could not compare this with the alter population which did not have the same requirement

Values shown are *N* (%) or mean ± SD. *p* values for continuous variables were determined by paired *t* tests; *p* values for categorical variables were determined by Chi-square tests

### Consistency of Index-Alter Reports

Table 2 reports the concordance and discordance of index and alter reports of prevention advocacy on HIV testing, condom use, engaging in HIV care, taking ARVs and PrEP use. There was nearly no discussion of PrEP and due to the high symmetry of responses for HIV care engagement, and ARV adherence, no further statistics were computed for these variables after the conditional probabilities, the kappa and McNemar statistics. Thus, we moved forward with further analyses on condom use and HIV testing advocacy only. Because the prevalence of condom use and HIV testing advocacy responses was skewed [37], we calculated conditional probability indices, which indicated fair concordant agreement for condom use (CP + = 0.23) and poor concordant agreement for HIV testing (CP + = 0.10).

**Table 2:** Concordance of index and alters’ reported prevention advocacy

	Agreement *n* (%)	Kappa^a^	CP + ^b^	McNemar Statistic^c^ (*p*-value)
Talked about HIV Testing				
Both report yes	51 (18.3)	0.12	0.1	14.55 (< 0.001)
Both report no	104 (37.3)			
Discordant	124 (44.5)			
Talked about Condom use				
Both report yes	58 (9.6)	0.16	0.23	2.54 (0.11)
Both report no	348 (58.1)			
Discordant	193 (32.2)			
Talked about engaging in HIV Care				
Both report yes	46 (26.5)	0.13	0.08	1.35 (0.25)
Both report no	52 (30.1)			
Discordant	75 (43.4)			
Talked about taking ARVs				
Both report yes	86 (49.7)	0.21	0.15	0.96 (0.33)
Both report no	27 (15.6)			
Discordant	60 (34.6)			
Talked about PrEP use				
Both report yes	1 (0.29)	0.02	0.003	7.36 (0.007)
Both report no	307 (89.2)			
Discordant	36 (10.5)			

^a^Because each index participant talked with multiple alters, the cluster bootstrap method was used to calculate the kappa statistic

^b^Positive conditional probability index—see definition in “Methods” section

^c^Obuchowski chi-square statistic and *p*-value which adjusts for clustering on index participant

McNemar’s tests further revealed significant differences for agreement indicating that fewer indexes versus alters said “yes” to HIV testing prevention advocacy occurring (30.5% vs. 50.5%, respectively; ${\chi }^{2}$=25.3, *p*<0.001).

### Condom Use Regression Analyses

#### Factors Associated with Condom Use Prevention Advocacy Concordance

We employed multinomial logistic regression to investigate factors associated with concordance between indexes and alters in terms of condom use prevention advocacy (Table 3). Being a romantic partner [OR = 3.50; 95% CI (1.22, 10.06), *p* = 0.02], and the index being 10 years younger than the alter [OR = 0.23; 95% CI (0.06, 0.81]; *p* = 0.02) were associated with concordant index-alter prevention advocacy reporting. However, in instances where only the alter reported advocacy and the index did not, significant factors included dyadic gender composition [male index to male alter prevention advocacy, OR = 2.41; 95% CI (1.11, 5.23), *p* = 0.02]; female index to male alter prevention advocacy, OR = 2.35; 95% CI (1.22, 4.53), *p* = 0.01], and when the alter was a child [OR = 0.19; 95% CI (0.07, 0.51), *p* < 0.001] or a romantic partner [OR = 0.31; 95% CI (0.10, 0.94), *p* = 0.03].

**Table 3:** Multinomial logistic regression models assessing factors associated with condom use prevention advocacy

Description of Category	Alter reports advocacy but index does not	Index reports advocacy but alter does not	Both index and alter report advocacy occurred
Independent Variables	OR (CI)	OR(CI)	OR(CI)
Sex			
Male Index/Male Alter	2.41 (1.11, 5.23)*	2.18 (0.83, 5.76)	2.49 (0.87, 7.15)^+^
Female Index/Female Alter	Ref	Ref	Ref
Male Index/Female Alter	0.92 (0.47, 1.78)	1.15 (0.45, 2.90)	0.72 (0.24, 2.17)
Female Index/Male Alter	2.35 (1.22, 4.53)**	1.77 (0.95, 3.31)^+^	2.06 (0.86, 4.93)
Age			
Index 10yrs > = than Alter	1.30 (0.69, 2.42)	1.24 (0.61, 2.42)	1.17 (0.44, 3.09)
Alter 10yrs > = than Index	0.39 (0.17, 0.92)*	0.29 (0.08, 0.99)*	0.23 (0.06, 0.81)*
Index is within 10yrs of alter	Ref	Ref	Ref
Relationship status			
Child	0.19 (0.07, 0.51)***	1.08 (0.36, 3.20)	0.57 (0.15, 2.13)
Other relative	0.61 (0.28, 1.33)	1.32 (0.49, 3.51)	0.87 (0.24, 3.23)
Sibling	0.98 (0.56, 1.74)	1.25 (0.56, 2.78)	1.58 (0.65, 3.79)
Romantic Partner	0.31 (0.10, 0.94)*	1.65 (0.63, 4.34)	3.50 (1.22, 10.06)*
Friend	Ref	Ref	Ref
Frequency of contact			
About 1–3 times a month	1.31 (0.54, 3.18)	0.31 (0.06, 1.53)	0.78 (0.13, 4.58)
About 1–2 times a week	0.83 (0.38, 1.93)	0.72 (0.29, 1.77)	1.24 (0.31, 4.85)
About 3–5 times/week	Ref	Ref	Ref
Everyday or nearly everyday	1.65 (0.88, 3.06)	1.38 (0.68, 2.78)	2.26 (0.81, 6.35)

^*+*^*p* < 0.10; ^*^p < 0.05; ^**^p < 0.01; ^***^p < 0.001

#### Concordance of Perceived Presence of Condom Use Advocacy as a Predictor of Alter Condom Use

A total of 97 alters reported using condoms the last time they had intercourse with their main partners, while 358 reported not using condoms. As shown in Table 4, regression findings indicated that alters had increased odds of using condoms with their main partner when both the index and the alter reported prevention advocacy occurred, compared to dyads where neither reported advocacy [OR = 3.90; 95% CI (2.00,7.59); *p* < 0.001]. In addition, alters had increased odds of using condoms when both the index and the alter reported prevention advocacy occurred, compared to dyads where only the index reported such advocacy [OR = 3.71; 95% CI (1.35,10.18); *p* = 0.01]; and when only the alter reported advocacy occurring, compared to when neither index nor alter confirmed advocacy [OR = 2.23; 95% CI (1.19, 4.15,); *p* = 0.01], or only the index reported advocacy [OR = 2.66; 95% CI (1.03, 6.93); *p* = 0.04]. Neither age nor sex homophily were found to be significant covariates of alter condom use. Relationship types were excluded from the models presented in Table 4 due to overfitting the number of independent variables in the model when the sample size was small. However, when relationship status was analyzed independently, we found significant associations for spouse, child, and other relative categories.

**Table 4:** Concordance of perceived presence of condom use prevention advocacy (PA) as associated with the dependent variable alter condom use

Type of concordance	Model 1: Concordance reporting of PA when compared to no reporting of PA	Model 2: Concordance reporting of PA when compared to only index reporting of PA	Model 3: Concordance reporting of PA when compared to only alter reporting of PA	Model 4: Index reporting of PA compared to no reporting of PA	Model 5: Alter only reporting of PA compared to no reporting of PA	Model 6: Index reporting of PA compared to only alter reporting of PA
Independent Variables	OR (CI)	OR (CI)	OR (CI)	OR (CI)	OR (CI)	OR (CI)
PA Concordance	3.90 (2.00, 7.59)***	3.71 (1.35, 10.18)**	1.64 (0.72, 3.73)	0.84 (0.36, 1.94)	2.23 (1.19, 4.15)*	2.66 (1.03, 6.93)*
Sex						
Male Index/Male Alter	1.90 (0.69, 5.20)	0.54 (0.09, 3.01)	0.84 (0.24, 2.96)	1.35 (0.46, 3.94)	1.21 (0.51, 2.86)	0.56 (0.16, 1.99)
Female Index/Female Alter	Ref	Ref	Ref	Ref	Ref	Ref
Male Index/Female Alter	1.26 (0.56, 2.95)	1.97 (0.51, 7.59)	1.12 (0.37, 3.36)	1.22 (0.50, 2.94)	1.06 (0.47, 2.39)	0.99 (0.29, 3.31)
Female Index/Male Alter	1.08 (0.51, 2.28)	2.38 (0.75, 7.55)	1.40 (0.52, 3.79)	1.41 (0.66, 2.97)	1.13 (0.55, 2.33)	2.08 (0.78, 5.53)
Age						
Index 10yrs > = than Alter	0.99 (0.54, 1.83)	0.37 (0.12, 1.13)+	1.41 (0.61, 3.23)	1.06 (0.54, 2.09)	1.68 (0.91, 3.11)+	1.48 (0.56, 3.94)
Alter 10yrs > = than Index	0.72 (0.23, 2.22)	0.87 (0.11, 7.16)	0.68 (0.14, 3.37)	0.77 (0.24, 2.48)	0.87 (0.29, 2.54)	0.87 (0.15, 4.87)
Index is within 10yrs of alter	Ref	Ref	Ref	Ref	Ref	Ref

^*+*^*p* < 0.10; ^*^p < 0.05; ^**^p < 0.01; ^***^p < 0.001

### HIV Testing Regression Analyses

No factors were associated with HIV testing prevention advocacy concordance in multinomial logistic regressions (Table 5). Moreover, 392 individuals had undergone HIV testing within the past 6 months, and 207 had not; neither concordance nor discordance in reporting HIV prevention advocacy among index-alter dyads were predictors of alter HIV testing (Table 6).

**Table 5:** Multinomial logistic regression models assessing factors associated with HIV testing prevention advocacy

Description of Category	Alter reports advocacy but index does not	Index reports advocacy but alter does not	Both index and alter report advocacy occurred
Independent Variables	OR (CI)	OR(CI)	OR(CI)
Sex			
Male Index/Male Alter	1.19 (0.44, 3.23)	1.89 (0.40, 8.98)	1.78 (0.63, 5.06)
Female Index/Female Alter	Ref	Ref	Ref
Male Index/Female Alter	0.38 (0.11, 1.32)	1.49 (0.39, 5.74)	0.95 (0.23, 3.91)
Female Index/Male Alter	0.71 (0.29, 2.28)	1.12 (0.35, 3.61)	0.82 (0.29, 2.28)
Age			
Index 10yrs > = than Alter	0.93 (0.41, 2.12)	0.75 (0.17, 3.26)	0.40 (0.11, 1.49)
Alter 10yrs > = than Index	0.41 (0.12, 1.39)	1.00 (0.23, 4.35)	0.18 (0.03, 1.26)+
Index is within 10yrs of alter	Ref	Ref	Ref
Relationship status			
Child	1.42 (0.46, 4.33)	5.41 (0.69, 42.13)	2.80 (0.55, 14.06)
Other relative	0.57 (0.15, 2.11)	1.69 (0.25, 11.28)	0.64 (0.09, 4.11)
Sibling	0.65 (0.29, 1.46)	1.14 (0.25, 5.10)	0.54 (0.17, 1.66)
Romantic Partner	2.92 (0.44, 19.09)	6.53 (0.65, 65.13)	8.48 (1.25, 57.23)+
Friend	Ref	Ref	Ref
Frequency of contact			
About 1–3 times a month	0.90 (0.21, 3.87)	0.62 (0.07, 4.95)	0.15 (0.01, 2.01)
About 1–2 times a week	1.14 (0.42, 3.05)	0.91 (0.21, 3.96)	0.21 (0.04, 1.27)+
About 3–5 times/week	Ref	Ref	Ref
Everyday or nearly everyday	2.03 (0.82, 5.01)	1.49 (0.5, 4.85)	1.21 (0.37, 3.89)

^*+*^*p* < 0.10

**Table 6:** Concordance of perceived presence of condom use prevention advocacy (PA) associated with the dependent variable alter HIV testing (last 6 months)

Type of concordance	Model 1: Concordance reporting of PA when compared to no reporting of PA	Model 2: Concordance reporting of PA when compared to only index reporting of PA	Model 3: Concordance reporting of PA when compared to only alter reporting of PA	Model 4: No reporting of PA compared to only index reporting of PA	Model 5: No reporting of PA compared to only alter reporting of PA	Model 6: Index reporting of PA compared to only alter reporting of PA
Independent Variables	OR (CI)	OR (CI)	OR (CI)	OR (CI)	OR (CI)	OR (CI)
PA Concordance	0.66 (0.29, 1.49)	0.72 (0.19, 2.66)	1.16 (0.49, 2.73)	0.86 (0.33, 2.20)	0.57 (0.28, 1.18)	0.80 (0.27, 2.32)
Sex						
Male Index/Male Alter	0.64 (0.25, 1.62)	0.49 (0.09, 2.52)	1.86 (0.59, 5.84)	0.57 (0.19, 1.72)	1.32 (0052, 3.32)	2.05 (0.59, 7.02)
Female Index/Female Alter	Ref	Ref	Ref	Ref	Ref	Ref
Male Index/Female Alter	0.29 (0.12, 0.72)	0.14 (0.03, 0.66)**	0.37 (0.94, 1.49)	0.36 (0.15, 0.91)	0.53 (0.22, 1.29)	0.60 (0.17, 2.13)
Female Index/Male Alter	0.33 (0.12, 0.82)	0.55 (0.14, 2.16)	0.68 (0.26, 1.77)	0.48 (0.19, 1.17)	0.59 (0.26, 1.34)	1.18 (0.42, 3.32)
Age						
Index 10yrs > = than Alter	0.61 (0.26, 1.45)	0.32 (0.06, 1.61)	0.77 (0.33, 1.84)	0.77 (0.32, 1.84)	0.94 (0.46, 1.93)	0.97 (0.39, 2.41)
Alter 10yrs > = than Index	1.16 (0.39, 3.45)	3.40 (0.74, 15.68)	1.80 (0.31, 10.62)	1.59 (0.62, 4.10)	1.35 (0.53, 3.42)	2.44 (0.66, 8.89)
Index is within 10yrs of alter	Ref	Ref	Ref	Ref	Ref	Ref

^*+*^*p* < 0.10; ^*^p < 0.05; ^**^p < 0.01; ^***^p < 0.001

## Discussion

Findings of the present study suggest a clear link between condom use prevention advocacy concordance and alter condom use behavior in our sample. While previous studies [4, 38] have recognized the significance of advocacy in HIV prevention, our study stands out as one of the first to explore and underscore the significance of concordance perception of the presence of advocacy on the part of both the advocate and the recipient or target of the advocacy, as we have demonstrated its relationship to condom use by the recipient.

In our examination of the concordance of prevention advocacy reports between alters and indexes, more alters than indexes reported advocacy discussions occurring for condom use and HIV testing, indicating the need to explore underlying reasons for this asymmetry in the perception of the presence of advocacy discussions, or alternatively the understanding of what constitutes advocacy. One possible hypothesis could be that alters, who may perceive themselves as more vulnerable or responsible for their own health outcomes, might be more inclined to initiate or recall these advocacy discussions compared to indexes. Moreover, when only the alter reported presence of condom use advocacy, this too was associated with alter condom use. This suggests that while concordance may be important, alters’ perceptions of advocacy may be the strongest catalysts for behavior change. Interventions are needed that train indexes in communication skills that clearly convey when advocacy is occurring.

We also explored factors related to concordant perception of advocacy, finding that age was associated with condom use advocacy concordance. When the alter was older, there was a lower likelihood of both index and alter reporting presence of condom advocacy. This could be an indication that in some African cultures, respect for elders and the hierarchical dynamics among different age groups in certain African cultures, significantly impact communication patterns, particularly concerning sensitive topics like sexual health. Age shapes communication styles and decision-making processes, and within the realm of sexual health discussions, this hierarchical structure may obstruct open dialogue or limit the options when considering who is appropriate to give advice. Younger individuals might find it challenging to broach such topics with older individuals due to the perceived social disparities in their relationship. Offering condom use advocacy to someone older might be perceived as a breach of respect or a challenge to traditional norms, discouraging both parties from engaging in such discussions. Thus, interventions or educational approaches on sensitive topics like condom use need to be adapted and tailored to align with the cultural dynamics and respect for age-related hierarchies in Africa.

Type of relationship between index and alter also was associated with concordance of perceived advocacy. For both condom use and HIV testing prevention advocacy concordances, findings revealed higher odds of concordance on HIV testing and condom use prevention advocacy when the alter was a romantic partner. In the case of romantic partners, intimate involvement in the relationship might lead them to assume that such conversations had occurred, emphasizing the importance of clear and explicit communication regarding condom use.

### Limitations

Overall, the findings of this study provide insights into the factors influencing concordance of perceived advocacy with implications for tailored and culturally sensitive HIV prevention interventions in sub-Saharan Africa. However, the cross-sectional design of the data prevents us from making causal inferences between the advocacy received and targeted HIV prevention behaviors. We relied on self-reported data; thus, it is possible that participants answered questions in a socially desirable way or that their responses were subject to recall bias. Furthermore, our approach to assessing concordance involved querying the index about conversations with the alter regarding specific behavioral topics (like condom use or HIV testing) and asking the alter if the index had engaged in similar discussions about these HIV-related health behaviors. However, this method presents a limitation because never asked the respondents what they considered to be advocacy. Thus, we do not know whether the participants specifically perceived the occurrence of a discussion as prevention advocacy or simply a discussion of an HIV-related topic, which could affect the level of concordance observed. Lastly, this study had a small sample size and a small geographic footprint, and was restricted to indexes who were PLWH in HIV care, all of which limits generalizability of findings to prevention advocacy conducted by PLWH more generally. Future mixed methods research, including surveys with larger and more representative samples as well as qualitative research to understand the nature of alter-index concordance and discordance, would be beneficial for a comprehensive examination of prevention advocacy concordance and health behaviors.

## Conclusion

These findings provide insights into the determinants of dyadic agreement in perceived occurrence of prevention advocacy, offering potential avenues for enhancing the effectiveness of advocacy interventions. The results can help to elucidate the dynamics of peer-based health advocacy in social networks, highlighting the importance of gender dynamics, and relationship type, in shaping concordance in what is perceived to be advocacy, which in turn may influence the impact of advocacy on the target HIV protective behavior. Understanding these dynamics provides valuable information for how to design targeted approaches, maximizing the impact of advocacy efforts on health behavior outcomes within diverse social contexts.
